# Recombinant human milk fat globule-EGF factor VIII (rhMFG-E8) as a therapy for sepsis after acute exposure to alcohol

**DOI:** 10.1186/s10020-019-0118-x

**Published:** 2019-11-20

**Authors:** Wayne W. Chaung, Max Brenner, Hao-Ting Yen, Mahendar L. Ochani, Asha Jacob, Ping Wang

**Affiliations:** 1 TheraSource LLC, 350 Community Dr, Manhasset, NY 11030 USA; 2 Center for Immunology and Inflammation, The Feinstein Institutes for Medical Research, 350 Community Dr, Manhasset, NY 11030 USA; 3 Department of Molecular Medicine, Zucker School of Medicine at Hofstra/Northwell, Manhasset, NY 11030 USA; 4 Department of Surgery, Zucker School of Medicine at Hofstra/Northwell, Manhasset, NY 11030 USA

**Keywords:** Alcohol, Sepsis, CLP, rhMFG-E8, Survival, Therapy

## Abstract

**Background:**

Alcohol intake predisposes to infections and sepsis. Alcohol and sepsis inhibit the expression of milk fat globule epidermal growth factor-factor VIII (MFG-E8), a glycoprotein essential for optimal efferocytosis, resulting in the release of proinflammatory molecules and increased sepsis severity. We previously reported that recombinant mouse (rm) MFG-E8 attenuates sepsis-induced organ injury in rats with acute alcohol intoxication. In order to develop a therapy that can be safely used in humans, we have produced recombinant human (rh) MFG-E8 and evaluated its efficacy to ameliorate sepsis after acute exposure to alcohol.

**Methods:**

We induced acute alcohol intoxication with a bolus injection of alcohol (1.75 g/kg BW) followed by an intravenous infusion of 300 mg/kg/h alcohol for 10 h. Sepsis was then induced by cecal ligation and puncture (CLP). At -10, 0, and 10 h relative to CLP, rats received MFG-E8 or vehicle (albumin) intravenously. Animals were euthanized at 20 h after CLP for blood and tissue collection. Additional groups of animals were used for a survival study.

**Results:**

Compared to vehicle, rhMFG-E8 treatment ameliorated blood levels of proinflammatory cytokines (% improvement: TNF-α 49.8%, IL-6 34.7%) and endotoxin (61.7%), as well as of transaminases (AST 36.2%, ALT 40.1%) and lactate (18.4%). Rats treated with rhMFG-E8 also had a significant histological attenuation of the acute lung injury, as well as a reduction in the number of apoptotic cells in the thymus (43.4%) and cleaved caspase 3 (38.7%) in the spleen. In addition, rhMFG-E8 improved the 10-day sepsis survival rate from 45 to 80%

**Conclusion:**

rhMFG-E8 significantly ameliorated sepsis in rats with acute alcohol exposure, demonstrating rhMFG-E8’s potential to be developed as an effective therapy for sepsis in alcohol abusers.

## Introduction

Sepsis, defined as a life-threatening organ dysfunction caused by a dysregulated host response to infection (Singer et al. 2016), results in an estimated 5.3 million deaths worldwide annually (Fleischmann et al. 2016). Known effective treatments include early administration of empirical antibiotics, infection source control whenever possible, fluid resuscitation, and supportive measures to compensate for organ dysfunction such as circulatory collapse, respiratory failure, and renal injury. Yet, sepsis mortality rates remain as high as 26% (Fleischmann et al. 2016), revealing an urgent medical need for novel and effective treatments for septic patients. In the last 50 years, however, clinical trials have repeatedly failed to identify effective new treatments for sepsis, leading to the somber sobriquet “the graveyard of pharmaceutical discovery” (Lee 2018). One reason proposed for the lack of efficacy in clinical trials is including septic patients with diverse predisposing conditions, different forms of infection, and heterogeneous clinical presentations (Vincent and Sakr 2019; Seeley and Bernard 2016). As such, one new strategy has been to identify endophenotypes representing more homogeneous subgroups of septic patients who are more likely to share pathogenic mechanisms and to respond to a given therapeutic intervention (Mebazaa et al. 2016). One potential sepsis endophenotype is sepsis in alcohol abusers (Burnham et al. 2003).

Alcohol abuse is highly prevalent. According to the 2016 National Survey on Drug Use and Health, 25.5% of Americans ages 18 and older (62.6 million) report having engaged in binge drinking in the past month (Substance Abuse and Mental Health Services Administration 2017). Excessive alcohol drinking is a major risk factor for developing alcohol use disorders (AUD) (Vincent and Sakr 2019). In addition to their well-known adverse effects on the hepatic, nervous, and cardiovascular function, AUD is also associated with increased morbidity and mortality due to infection (Trevejo-Nunez et al. 2015; Probst et al. 2018; Waldschmidt et al. 2008; Bird and Kovacs 2008; Ness et al. 2008; Aloman et al. 2007; Cook 1998). Alcohol impairs the immune system, rendering alcohol abusers more susceptible to a wide range of infectious diseases, including bacterial sepsis (Trevejo-Nunez et al. 2015; Jin et al. 2017; Klingensmith et al. 2019). As a result, serious bacterial infections in patients with AUD are associated with increased tissue damage, higher morbidity, and increased mortality (Moss 2005; Gustot et al. 2017; Simou et al. 2018; O'Brien Jr. et al. 2007; de Wit et al. 2007). These patients often develop complications such as delirium and liver cirrhosis, which also exacerbate mortality (Vincent and Sakr 2019). Despite the increased prevalence and severity of sepsis in patients with AUD, no specific treatments have yet been developed to ameliorate sepsis in alcohol abusers.

**Fig. 1:**
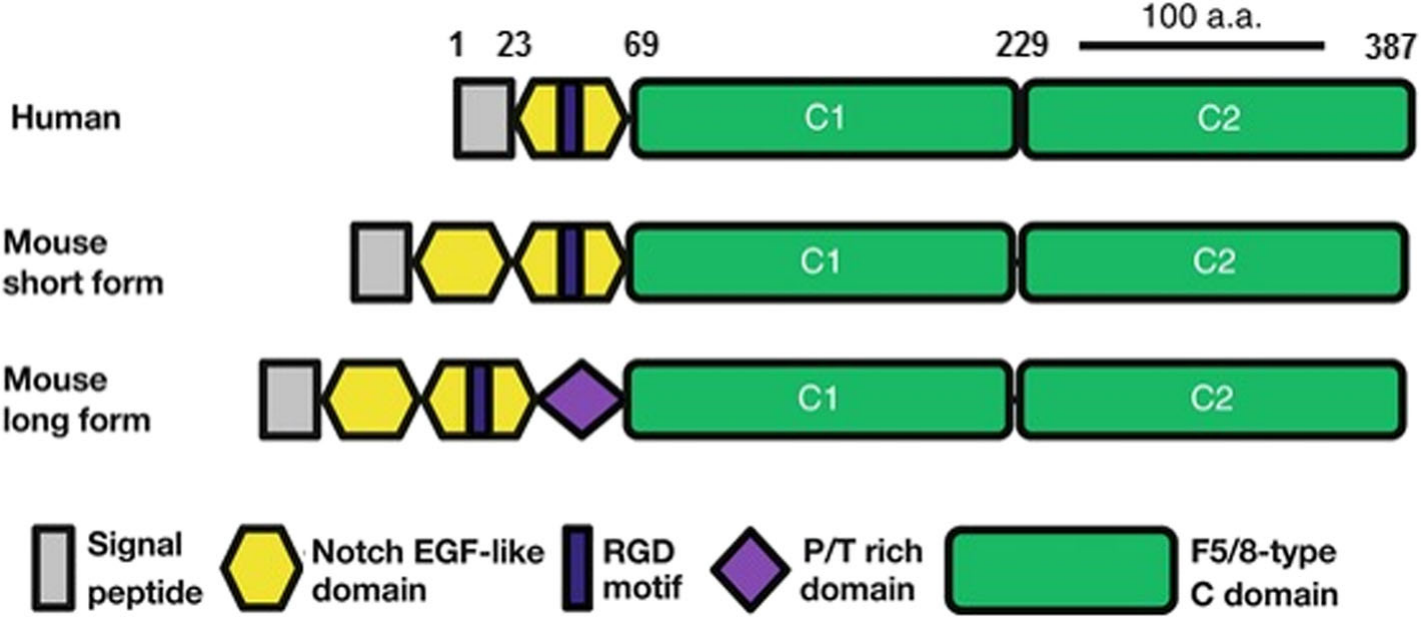
*Comparison of molecular structure and functional domains between human MFG-E8 and the long and short forms of mouse MFG-E8 (modified from (*Oshima et al. 2014*))*

Milk fat globule-epidermal growth factor-factor VIII (MFG-E8), also called lactadherin, is a secreted glycoprotein abundant in milk, including human breast milk (Yasueda et al. 2015; Sabha et al. 2018). We have shown that, among other functions such as anti-coagulant (Shah et al. 2012), anti-atherogenic (Miksa et al. 2009b), pro-extracellular matrix remodeling (Aziz et al. 2013) and enterotrophic activity (Bhatty et al. 2017), MFG-E8 has anti-inflammatory properties (Miksa et al. 2009a). MFG-E8 attenuates inflammation by promoting the phagocytic clearance of the dying cells termed efferocytosis (Hanayama et al. 2002; Aziz et al. 2011). MFG-E8 promotes efferocytosis by binding to phosphatidylserine (PS) on the cell membrane of apoptotic cells via its C-terminal F5/8-type discoidin domains while also binding to α_v_β_3_ integrin on phagocytes via its RGD (arginine-glycine-aspartic acid) motif in the N-terminal EGF-like domain (Fig. 1) (Oshima et al. 2014). We have previously shown that sepsis after acute alcohol exposure downregulates MFG-E8 expression, and that the exogenous administration of recombinant mouse (rm) MFG-E8 attenuates organ injury, systemic inflammation and mortality associated with this model (Wu et al. 2010; Chaung et al. 2008). However, immunogenicity precludes the use of animal proteins in humans. Therefore, we have expressed, purified and characterized recombinant human (rh) MFG-E8 protein (Qiang et al. 2011). Unlike its mouse orthologue, human MFG-E8 is a 387-amino acid 45-KDa protein with only one N-terminal EGF-like domain and no proline/threonine rich domain (Fig. 1), with an amino acid homology of 63% with mouse MFG-E8 (Sabha et al. 2018; Oshima et al. 2014).

In this study, we evaluated the potential benefit of using rhMFG-E8 to ameliorate sepsis associated with alcohol intoxication. We show that rhMFG-E8 decreased proinflammatory cytokines, endotoxemia, organ damage, acute lung injury, and apoptotic cells in septic rats with acute alcohol intoxication, and increased the survival rate of septic rats with acute alcohol exposure. These findings demonstrate that rhMFG-E8 can be developed as an effective therapy for sepsis associated with alcohol abuse.

## Materials and methods

### Experimental animals

Adult male Sprague-Dawley rats (200–250 g) purchased from Charles River Laboratories were housed in a humidity- and temperature-controlled room with a 12-h light cycle for at least 1 week. In order to minimize the total cecal content, rats were fasted overnight but were allowed water ad libitum before surgery. All experiments were performed following the National Institutes of Health (NIH) guidelines, and all the projects were approved by the Institutional Animal Care and Use Committee (IACUC) of the Feinstein Institutes for Medical Research, Manhasset, NY.

### Model of acute alcohol exposure

Acute alcohol exposure was performed following Bautista’s modified method (Wu et al. 2010; Bautista 1998; Bautista and Spitzer 1999). Briefly, the jugular vein was cannulated with PE-50 catheter under general anesthesia with isoflurane. Rats then received a bolus injection of alcohol (1.75 g/kg BW in 1 ml normal saline) via the jugular venous catheter, followed by an intravenous (*iv*) infusion of 300 mg/kg/h of alcohol for 10 h (total dose 4.75 g/kg BW) administered without anesthesia by use of a specialized animal restraint that allowed free movement of the rat within the cage.

### Cecal ligation and puncture (CLP) model of sepsis

At the end of acute alcohol exposure, rats were subjected to cecal ligation and puncture (CLP), as previously described (Qiang et al. 2013). Briefly, a 2-cm ventral midline abdominal incision was performed under isoflurane anesthesia. The cecum was exposed, ligated just distal to the ileocecal valve, and punctured twice with an 18-gauge needle. A small amount of cecal content was extruded. After the incision was closed in layers, the animals received fluid resuscitation with 30 ml/kg BW normal saline subcutaneously (*sc*). Rats subjected to sham surgery underwent the same procedure, except for the cecum perforation. At 20 h after surgery, all rats were euthanized for blood and tissue (i.e., the lungs, spleen and thymus) collection. Rats were euthanized at 20 h after CLP because at that time point systemic inflammation and tissue injury are detected, but mortality does not occur. This model represents severe sepsis and it has been extensively used by us and others to study the pathophysiology and therapeutic approaches (Wu et al. 2010; Cen et al. 2017; Miksa et al. 2006; Shah et al. 2012). To allow for a longer-term observation, the CLP model in survival studies was attenuated by surgical excision of the gangrenous cecum and irrigation of the peritoneal cavity with 30 ml warm sterile ringer’s lactate solution at 20 h after CLP. Changes in survival time were monitored and recorded over a 10-day period. Sham groups were not included in the survival study because sham-operated rats do not exhibit decreased survival.

### Production of rhMFG-E8

The rhMFG-E8 protein was produced by TheraSource (Manhasset, NY). The pET-28a(+) expression plasmid containing the His-tagged mature form of the human MFG-E8 (NM_005928.2; Leu24-Cys387) (GeneCopoeia, Inc., Germantown, MD) was transformed into *E. coli* BL21 (DE3) cells and high-yield colonies were selected, induced with IPTG, collected, and sonicated (Qiang et al. 2011). The sonicate was clarified by centrifugation and rhMFG-E8 was purified using metal affinity chromatography. Purity and authentication were assessed using SDS-PAGE and Western blotting, and the amino acid composition of the purified protein was confirmed by mass spectrum analysis at the Rockefeller University, NY. After phase-separation with Triton-X-114, the endotoxin content was < 0.01 EU/μg protein, one order of magnitude lower than the available commercial source. Using the pHrodo succinimidyl ester efferocytosis assay (Miksa et al. 2009b; Aziz et al. 2013), we observed that rhMFG-E8 increased the efferocytosis of apoptotic thymocytes by rat peritoneal macrophages by 3-fold, thus confirming that the rhMFG-E8 generated was biologically active.

### rhMFG-E8 administration

In order to determine the beneficial effects of MFG-E8 on sepsis after acute alcohol exposure, rats received 1-ml *iv* injections of normal saline containing either rhMFG-E8 (20 μg/kg BW per injection), rmMFG-E8 (positive control, R&D Systems, Minneapolis, MN; 20 μg/kg BW per injection) or control (human albumin, 20 μg/kg BW per injection) at the beginning of alcohol injection (-10 h), the beginning of CLP (0 h), and 10 h after CLP (10 h). Sham-operated animals were exposed to neither alcohol nor rhMFG-E8.

### Determination of serum levels of cytokines, endotoxin, liver enzymes, and lactate

The concentrations of TNF-α and IL-6 in the serum were quantified using commercially obtained enzyme-linked immunosorbent assay (ELISA) kits specific for rat TNF-α and IL-6 (BioSource International, Camarillo, CA). Serum endotoxin levels were measured using a limulus amebocyte lysate (LAL) kit (QCL-1000™, Lonza, Walkerville, MD). Serum levels of the liver enzymes aspartate aminotransferase (AST), alanine aminotransferase (ALT), and lactate were determined using colorimetric assay kits (Pointe Scientific, Canton, MI). All assays were performed according to instructions provided by the manufacturer. All samples were tested in duplicates.

### Lung histological analysis

Lung tissue sections were fixed in 10% formalin and embedded in paraffin, microsectioned into 5-μm sections, and stained with hematoxylin and eosin (H&E). Lung injury was evaluated by an investigator blinded for the study groups.

### Flow cytometry apoptosis assay

Apoptotic thymocytes were detected by flow cytometry analysis using a BD FACS Calibur cytometer. Briefly, cells from thymus were isolated, purified and resuspended in PBS at the concentration of 1 × 10^7^ cell/ml. After washing twice, cells were incubated with annexin V in a buffer containing propidium iodide (PI; “FITC annexin V apoptosis detection Kit”; BD Biosciences, cat. 556,547), and analyzed by flow cytometry. The majority of the cells isolated from sham group were viable and not undergoing apoptosis (annexin V negative and PI negative). We counted early apoptotic cells (annexin V positive and PI negative). Annexin V positive and PI positive cells are in late stage apoptosis or already necrotic, and were not included in the apoptotic cell counts.

### Cleaved caspase-3 protein activity assay

Spleen samples were lysed and homogenized in 1 ml of lysis buffer (10 mM TBS, 1 mM EDTA, 1 mM EGTA, 2 mM sodium orthovanadate, 0.2 mM PMSF, 2 μg /ml leupeptin, 2 μg /ml aprotinin, and 1% Triton X-100) for 30 min on ice and cleared by centrifugation at 12,000 g for 15 min at 4 °C. Caspase 3 is one of the critical enzymes for apoptosis and it is able to hydrolyze the peptide substrate Ac-DEVD-AMC to fluorescent 7-amino-4-methylcoumarin (AMC) moiety. Therefore, we measured the AMC fluorescence of freshly obtained spleen tissue (25 μg) cell lysate samples from each group along with AMC standard solutions (ranging from 50 nM to 6 μM). The amount of detectable AMC fluorescent signal was used to represent the caspase 3 activity to quantify apoptosis in our samples.

### Statistical analysis

Multiple group analysis was carried out using one-way analysis of variance (ANOVA). Differences between groups were further assessed by either the Student-Newman-Keuls test. Survival curves were analyzed by Kaplan-Meier method and log rank test. Difference in values were considered significant if *P* < 0.05.

## Results

### rhMFG-E8 attenuates serum levels of TNF-α, IL-6, and endotoxin in CLP after acute alcohol exposure

**Fig. 2:**
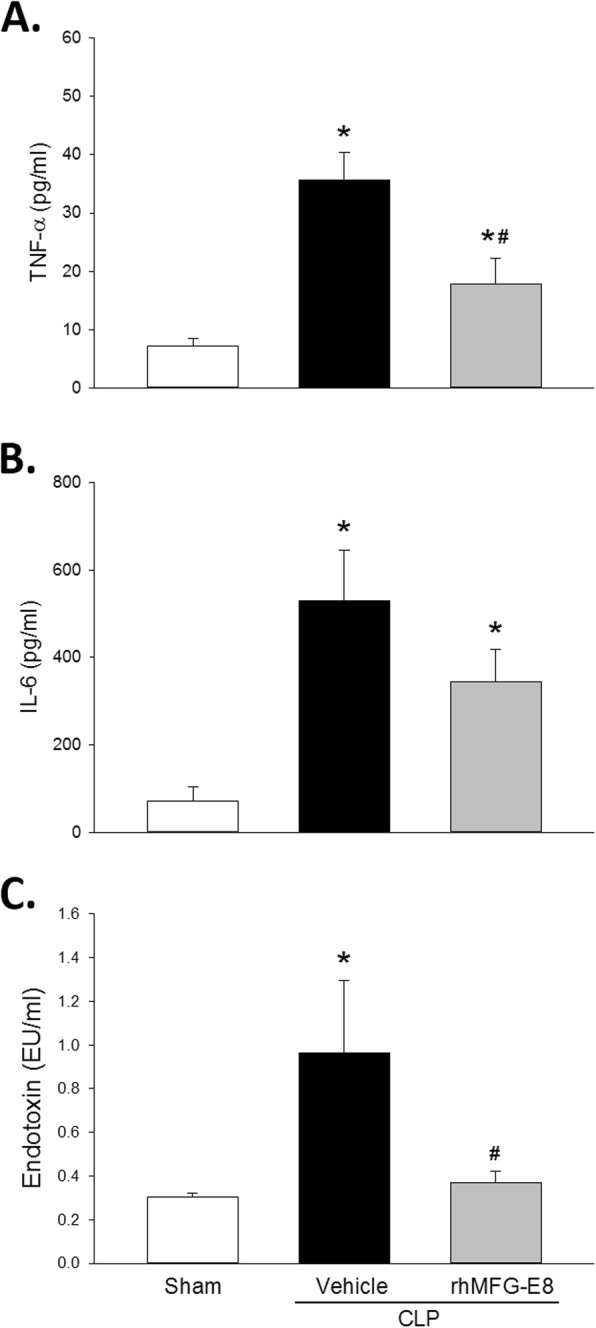
*rhMFG-E8 reduces systemic inflammation and bacteremia in CLP after acute alcohol exposure.* Levels of (**a**) serum TNF-α, (**b**) serum IL-6, and (**c**) serum endotoxin were increased rats subjected to CLP and treated with vehicle (human albumin) compared with sham surgery, and lower in rats subjected to CLP and treated with rhMFG-E8. *Prior to CLP, all rats were exposed to acute alcohol intoxication; means* *+* SE; n = 10–14 for sham and n = 9 for other experimental groups; ANOVA plus Student-Newman-Keuls method, * P < 0.05 vs. *Sham,* ^*#*^ *P < 0.05* vs. *Vehicle*

Serum levels of TNF-α and IL-6 are indicative of the immune system activation during sepsis. Compared with rats subjected to sham surgery after acute alcohol intoxication, the serum levels of TNF-α increased by 4.9-fold in rats subjected to CLP and treated with vehicle after acute alcohol intoxication (Fig. 2a). Animals treated with rhMFG-E8, however, had a significant decrease of 49.8% in the TNF-α levels (Fig. 2a). Similarly, compared with sham surgery, the serum levels of IL-6 were significantly elevated 7.5-fold in rats subjected to CLP and treated with vehicle after acute alcohol intoxication (Fig. 2b). Animals treated with rhMFG-E8 had a decrease of 34.7% in the IL-6 levels, though the reduction was not statistically significant (Fig. 2b). Serum endotoxin levels can be used as indicator of bacteremia. Compared with sham surgery after acute alcohol intoxication, the serum endotoxin levels were 2.8-fold higher in rats subjected to CLP and treated with vehicle after acute alcohol intoxication (Fig. 2c). Treatment with rhMFG-E8 significantly reduced serum endotoxin by 61.8% (Fig. 2c). These results indicate that rhMFG-E8 not only attenuated the immune system hyper activation, but also reduced the bacteremia in sepsis associated with acute alcohol exposure.

### rhMFG-E8 attenuates liver injury and lactate levels in CLP after acute alcohol exposure

**Fig. 3:**
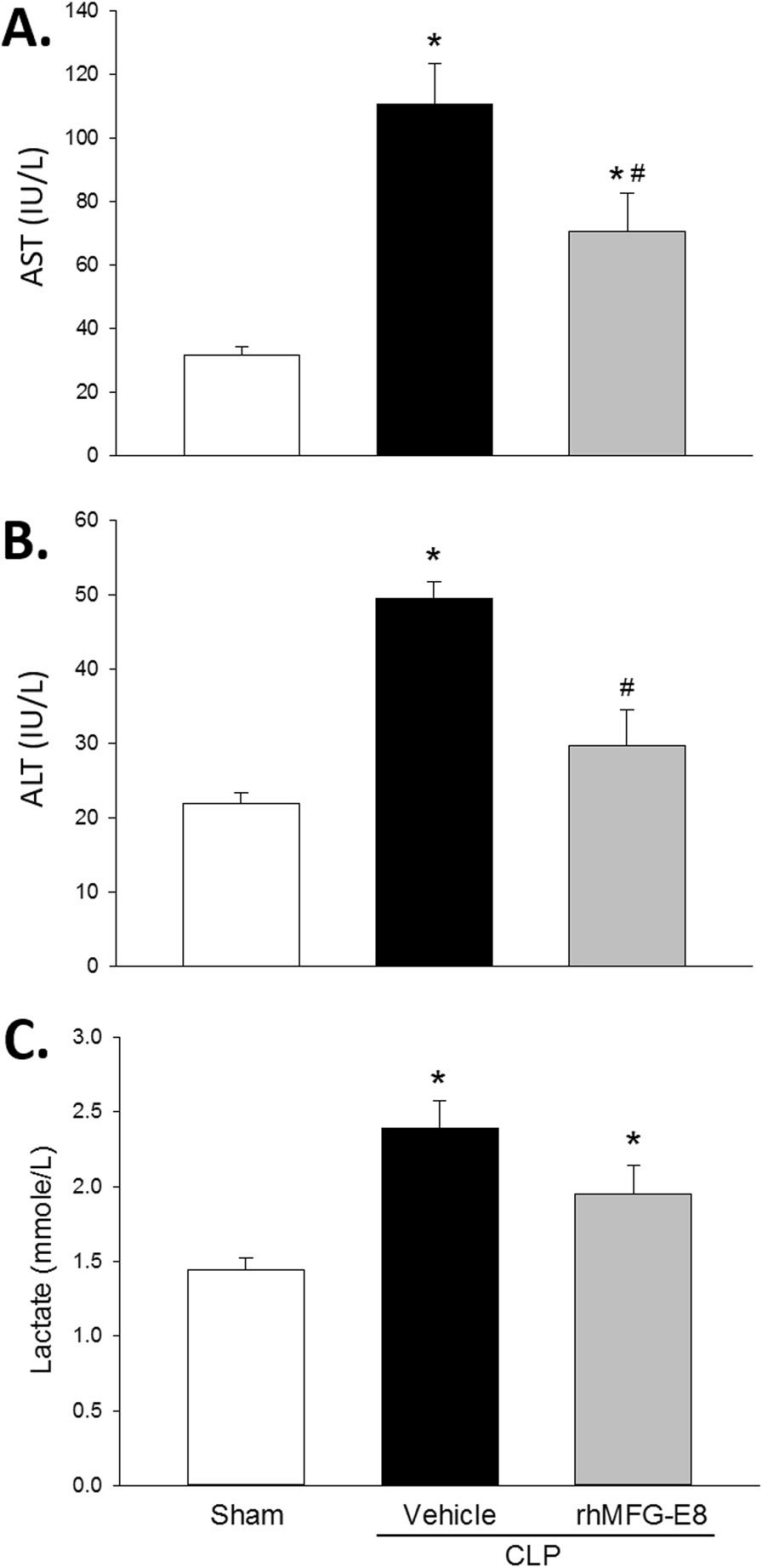
*rhMFG-E8 attenuates organ injury and hypoxia in CLP after acute alcohol exposure.* Serum levels of (**a**) AST, **b** ALT, and (**c**) lactate were increased rats subjected to CLP and treated with vehicle (human albumin) compared with sham surgery, and lower in rats subjected to CLP and treated with rhMFG-E8. *Prior to CLP, all rats were exposed to acute alcohol intoxication; means* *+* SE; n = 10–14 for sham and n = 9 for other experimental groups; ANOVA plus Student-Newman-Keuls method, * P < 0.05 vs. *Sham,* ^*#*^ *P < 0.05* vs. *Vehicle*

Aspartate aminotransferase (AST) and alanine aminotransferase (ALT) are often measured and used as biomarkers of liver damage. Compared with rats subjected to sham surgery after acute alcohol intoxication, the serum levels of AST increased by 3.5-fold in rats subjected to CLP and treated with vehicle after acute alcohol intoxication (Fig. 3a). Animals treated with rhMFG-E8, however, had a significant decrease of 36.2% in the AST levels (Fig. 3a). Similarly, compared with sham surgery, the serum levels of ALT were elevated 2.3-fold in rats subjected to CLP and treated with vehicle after acute alcohol intoxication (Fig. 3b), but animals treated with rhMFG-E8 had a significant decrease of 40.1% in the ALT levels (Fig. 3b). High levels of lactate, a product of glycolysis, are indicative of low tissue oxygenation. Compared with sham surgery after acute alcohol intoxication, the serum levels of lactate were 64.8% higher in rats subjected to CLP and treated with vehicle after acute alcohol intoxication (Fig. 3c). Treatment with rhMFG-E8 reduced the serum lactate levels by 18.4%, though the reduction was not statistically significant (Fig. 3c). These results indicate that rhMFG-E8 attenuated liver injury and improve tissue oxygenation in sepsis associated with acute alcohol exposure.

### rhMFG-E8 ameliorates acute lung injury in CLP after acute alcohol exposure

**Fig. 4:**
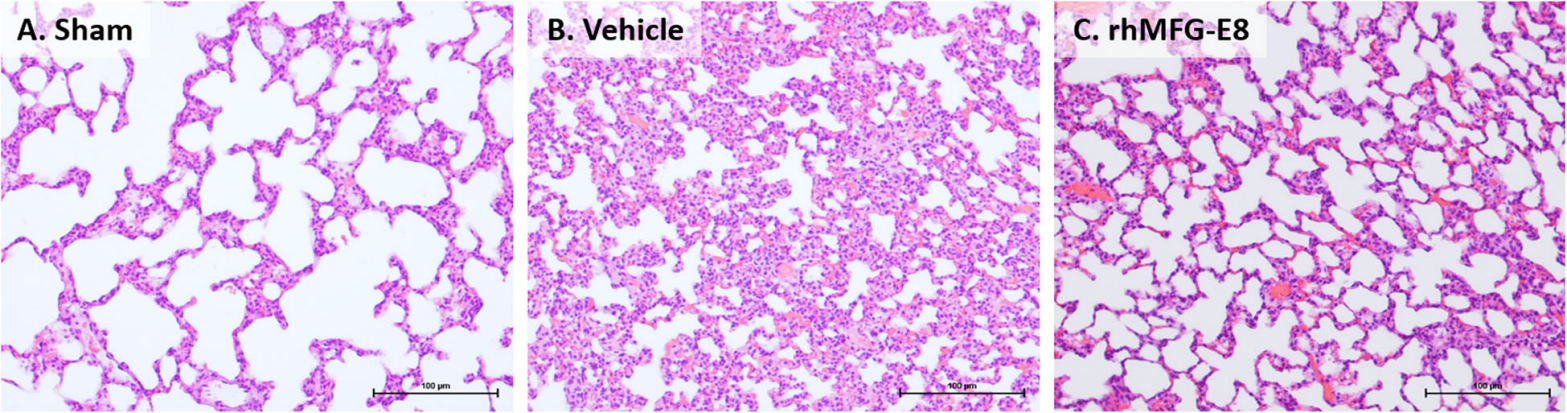
*rhMFG-E8 ameliorates acute lung injury in CLP after acute alcohol exposure.* While the lungs of (**a**) sham rats displayed a normal histological architecture, those of (**b**) CLP rats treated with vehicle had alveolar septal thickening with neutrophil infiltration and hyaline deposits. The lungs of (**c**) CLP rats treated with rhMFG-E8, however, had less severe histological lung injury. *Representative images of tissue sections with H&E staining: original magnification: 200×. Prior to CLP, all rats were exposed to acute alcohol intoxication; n = 10–14 for sham and n = 9 for other experimental groups*

The effects of rhMFG-E8 on acute lung injury associated with CLP after acute alcohol exposure were assessed histologically (Fig. 4a-c). Compared with sham, animals subjected to alcohol intoxication and CLP and treated with vehicle had alveolar septal thickening and hyaline deposits. The rats treated with rhMFG-E8, however, had significantly milder changes in the lung architecture and morphology compared with the vehicle group. These findings suggest that rhMFG-E8 is able to reduce acute lung injury in rats with sepsis after acute alcohol exposure.

### rhMFG-E8 reduces the number of apoptotic cells in tissues in CLP associated with acute alcohol exposure

**Fig. 5:**
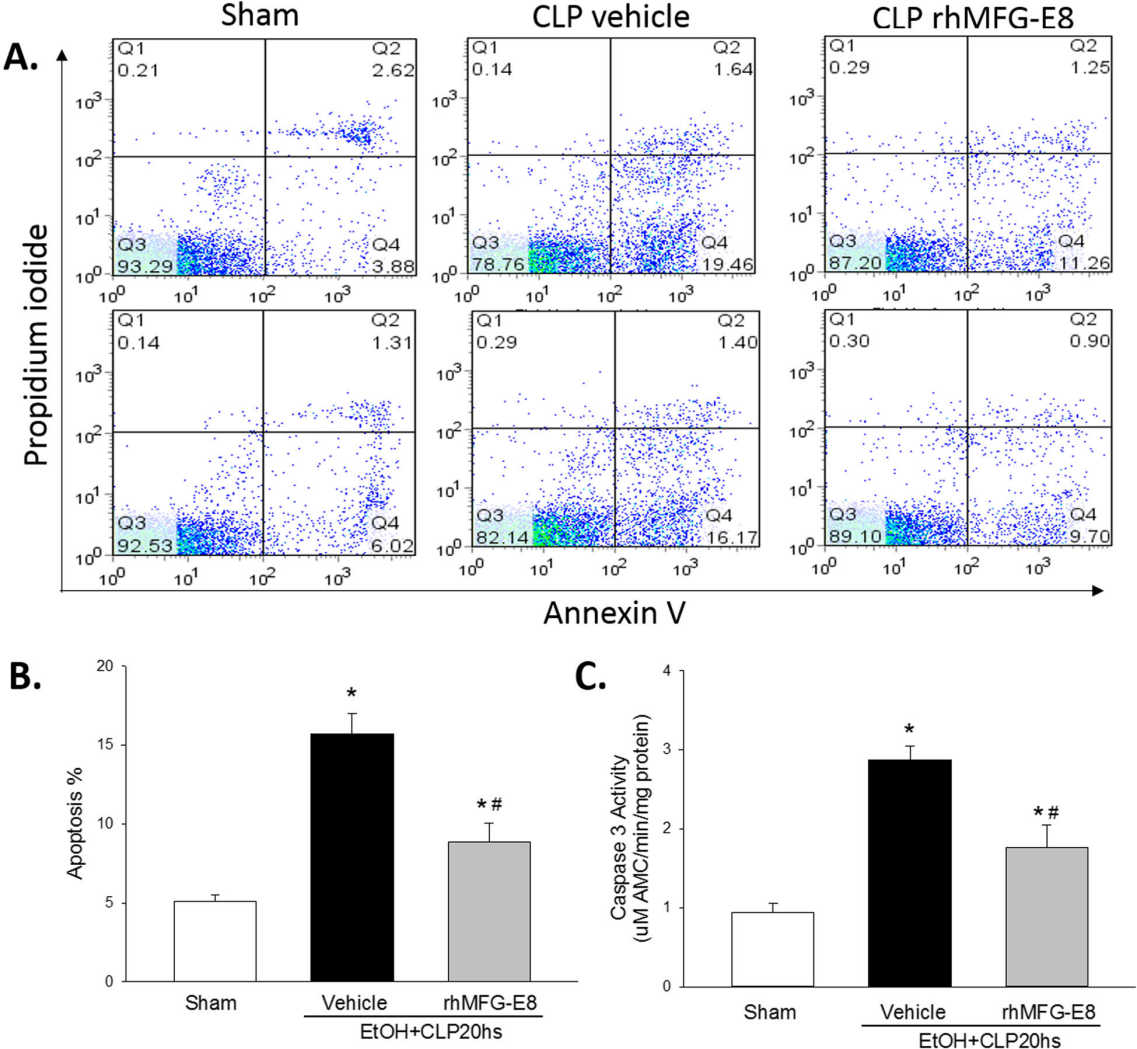
*rhMFG-E8 is associated with reduced apoptosis in CLP after acute alcohol exposure.* Early apoptotic cells in the thymus, (**a**) assessed by flow cytometry (lower right quadrant, Q4; representative image) (**b**) and quantified, and apoptosis in the (**c**) spleen, assessed using caspase 3 activity, were increased rats subjected to CLP and treated with vehicle (human albumin) compared with sham surgery, and lower in rats subjected to CLP and treated with rhMFG-E8. *Prior to CLP, all rats were exposed to acute alcohol intoxication; means* *+* SE; n = 10–14 for sham and n = 9 for other experimental groups; ANOVA plus Student-Newman-Keuls method, * P < 0.05 vs. *Sham,* ^*#*^ *P < 0.05* vs. *Vehicle*

MFG-E8 is known to facilitate the clearance of apoptotic cells by efferocytosis and, thus, decreases the number of detectable apoptotic events. To evaluate rhMFG-E8’s ability to reduce detectable apoptosis, we isolated thymocytes from all experimental groups (sham after acute alcohol exposure, CLP treated with vehicle after acute alcohol exposure, and CLP treated with rhMFG-E8 after acute alcohol exposure) and detected the rate of apoptotic cells using flow cytometry. Compared with rats subjected to sham surgery after acute alcohol intoxication, the rate of apoptotic thymocytes increased by 3.17-fold in rats subjected to CLP and treated with vehicle after acute alcohol intoxication (Fig. 5a, b). Animals treated with rhMFG-E8, however, had a significant decrease of 43.45% in the rate of apoptotic thymocytes (Fig. 5a, b). We also estimated the number of cells undergoing apoptosis by measuring caspase 3 activity in spleen samples. Compared with sham surgery after acute alcohol intoxication, the splenic caspase 3 activity was 3.0-fold higher in rats subjected to CLP and treated with vehicle after acute alcohol intoxication (Fig. 5c). Treatment with rhMFG-E8, however reduced the splenic caspase 3 activity by 38.7% (Fig. 5c). These results suggest that rhMFG-E8 effectively promoted the removal of apoptotic cells in sepsis associated with acute alcohol exposure.

### rhMFG-E8 improves the survival in CLP associated with acute alcohol exposure

**Fig. 6:**
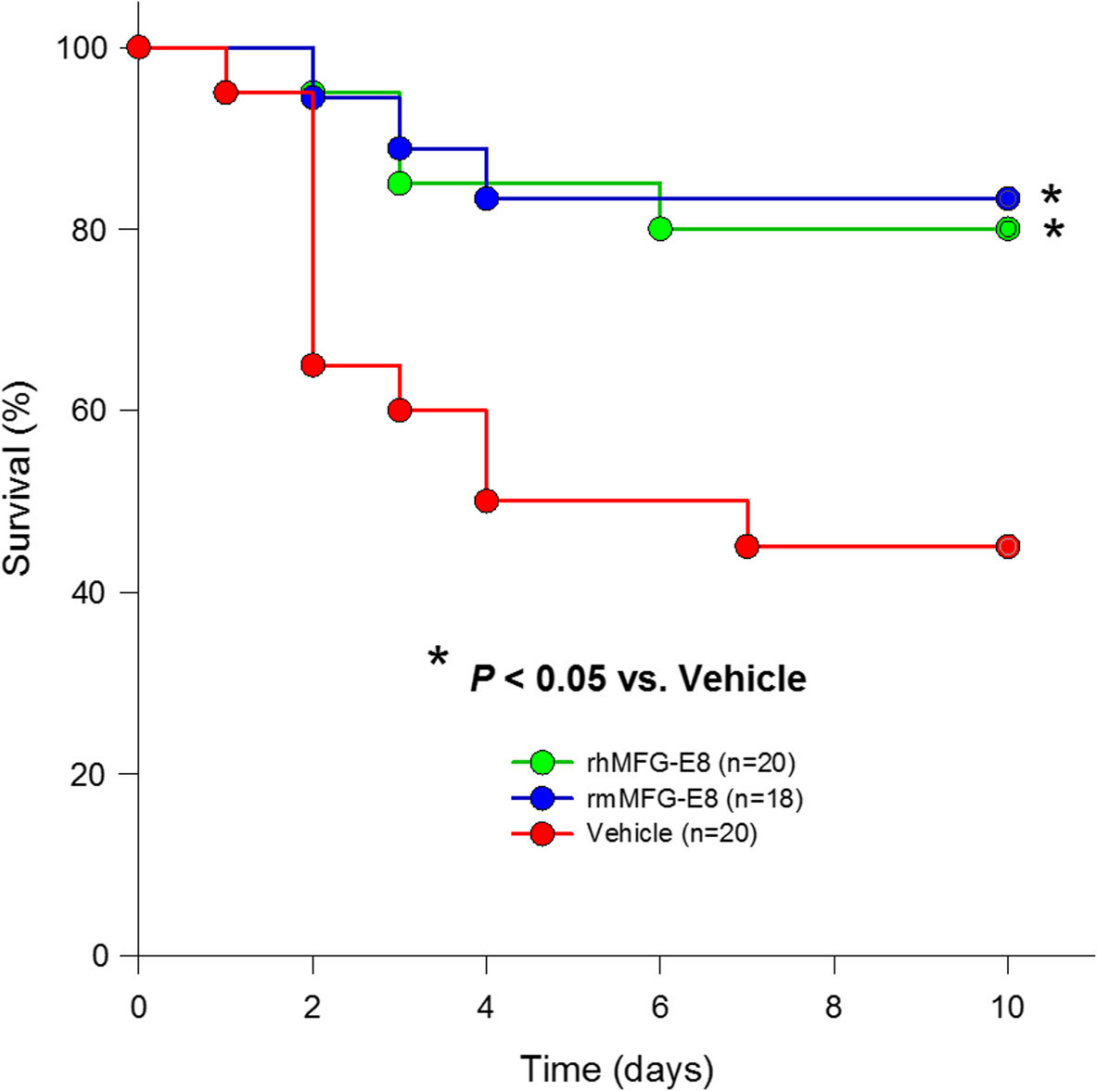
*rhMFG-E8 improves the survival rate in CLP after acute alcohol exposure.* The 10-day survival rate of rats subjected to CLP and treated with 20 μ/kg BW rhMFG-E8 was significantly higher than that of rats subjected to CLP and treated with vehicle (20 μ/kg BW human albumin). The survival benefit of animals treated with rhMFG-E8 was similar to that of animals treated with 20 μ/kg BW rmMFG-E8. Prior to CLP, all rats were exposed to acute alcohol intoxication; CLP was attenuated by surgical excision of the gangrenous cecum and peritoneal cavity irrigation at 20 h after CLP; n = 18 for rhMFG-E8 and n = 20 for other experimental groups); Kaplan-Meier plus log-rank test, * P < 0.05 vs. *Vehicle*

Finally, we determined whether rhMFG-E8’s survival benefit in CLP after acute alcohol intoxication was comparable to that of or commercially available rmMFG-E8. In order to decrease sepsis severity and allow for a survival study, CLP was attenuated by surgical excision of the gangrenous cecum and irrigation of the peritoneal cavity with sterile lactated ringer’s solution at 20 h after CLP. Rats subjected to CLP after acute alcohol intoxication and treated with vehicle (20 μ/kg BW human albumin) had a 10-day survival of 45% (Fig. 6). In contrast, rats treated with 20 μ/kg BW rhMFG-E8 had significantly improved 10-day survival rates of 80% (Fig. 6). Rats treated with commercially available 20 μ/kg BW rmMFG-E8, which was used as a positive control, also had a significantly improved survival of 83%. These results showed that differences in homology between rhMFG-E8 and rmMFG-E8 did not result in differences in efficacy, informed that the rat models are adequate for preclinical evaluation of rhMFG-E8, and reinforced the potential of developing rhMFG-E8 as a treatment for patients with sepsis associated with acute alcohol intoxication.

## Discussion

In the past decades, numerous clinical trials have failed to identify effective new treatments for sepsis as a single group. One rational alternative strategy for drug development in sepsis is, instead, to target therapeutic interventions to endophenotypes such as sepsis in alcohol abusers. Patients with a history of excessive alcohol use have higher rates of hospitalization, longer hospital stays, increased intensive care unit admissions, and more post-operative complications than those with no history of alcohol abuse (Probst et al. 2018; Waldschmidt et al. 2008; Bird and Kovacs 2008; Cook 1998). These patients also have increased frequency and severity of infection as a result of alcohol-induced immune dysfunction (Trevejo-Nunez et al. 2015; Cook 1998). Cells of the myelomonocytic lineage seem to be particularly sensitive to the immunomodulatory effects of ethanol (Ness et al. 2008; Aloman et al. 2007; Bautista 1998; Bautista and Spitzer 1999; Bhatty et al. 2017). For instance, Kupffer cells in alcoholic liver have inefficient phagocytosis and increased production of proinflammatory cytokines (McClain et al. 2005; Tilg et al. 1992), reactive oxygen species (Bautista 1998; Bautista and Spitzer 1999), and immune suppressive factors (Singer et al. 2016; Cook 1998; Tilg et al. 1992; Annoni et al. 1992). Other components of the immune system can also be impaired in patients with AUD (Jin et al. 2017). Consequently, excessive alcohol intake is associated with increased tissue damage, high morbidity and high mortality due to bacterial sepsis (Moss 2005; Gustot et al. 2017; Simou et al. 2018; O'Brien Jr. et al. 2007; de Wit et al. 2007).

We have previously shown that polymicrobial sepsis after acute alcohol exposure is associated with reduced expression of MFG-E8, and can be ameliorated by the administration of rmMFG-E8 (Wu et al. 2010), thus revealing a novel potential targeted therapy for sepsis in alcohol abusers. MFG-E8 is a secreted glycoprotein ubiquitously expressed and abundant in milk, including human breast milk (Yasueda et al. 2015; Sabha et al. 2018). Mainly produced by phagocytes such as macrophages and dendritic cells, MFG-E8 is necessary for their optimal clearance of dying (mostly apoptotic, but also autophagic, necrotic, necroptotic, pyroptotic) cells (Hanayama et al. 2002; Aziz et al. 2011). Sepsis is associated with a marked increase in the number of apoptotic cells, most notably lymphocytes but also enterocytes, cardiomyocytes, and kidney cells (Girardot et al. 2017; Luan et al. 2015; Kockara and Kayatas 2013; Chopra and Sharma 2007). If not removed by phagocytes, apoptotic cells undergo secondary necrosis releasing proinflammatory mediators that aggravate sepsis severity (Scaffidi et al. 2002; Aziz et al. 2019; Poon et al. 2014). Indeed, apoptosis inhibition has been proposed as a therapeutic strategy to attenuate sepsis (Hattori et al. 2010; Wesche-Soldato et al. 2007). Apoptotic and other dying cells can also be detected in numerous organs after alcohol exposure (Cho et al. 2018; Smith et al. 2015; Khanova et al. 2018; Liuzzi et al. 2018). Therefore, MFG-E8’s ability to promote the clearance of apoptotic cells makes it a logical choice to treat sepsis in alcohol abusers.

We have previously shown that MFG-E8 has anti-inflammatory effects in numerous preclinical models associated with increased levels of apoptosis (Wu et al. 2010; Cen et al. 2017; Shah et al. 2012; Ajakaiye et al. 2012; Cheyuo et al. 2012; Hansen et al. 2017a; Matsuda et al. 2013; Matsuda et al. 2011; Wu et al. 2012; Zhang et al. 2012; Hansen et al. 2017b). We have also shown that deficiency in MFG-E8 is detrimental to sepsis (Miksa et al. 2009a). Septic mice lacking MFG-E8 had two to three times more apoptotic cells in the spleen and a 60% higher mortality rate than septic wild-type mice (Miksa et al. 2009a). In addition, while sepsis alone caused a 49% decrease in MFG-E8 expression, sepsis after acute alcohol exposure produced 70% decrease in MFG-E8 (Oshima et al. 2014), suggesting that exposure to binge levels of alcohol predisposes to a much more severe deficit of MFG-E8 during sepsis. We have also shown that exogenous administration of rmMFG-E8 in sepsis after acute alcohol exposure provided protection from organ injury, systemic inflammation, and apoptosis (Wu et al. 2010). The current study shows that administration of rhMFG-E8 reduced apoptosis in the thymus and spleen in septic animals after acute alcohol expose. We have shown that MFG-E8 does not decrease the number of apoptotic cells through the direct modulation of apoptotic pathways but rather through the clearance of apoptotic cells (Miksa et al. 2006). Thus, one may speculate that the observed reduction in apoptosis with rhMFG-E8 is likely a consequence of efferocytosis rather than a direct decrease in apoptosis. Therefore, the promotion of efferocytosis with rhMFG-E8 offers an alternative strategy to apoptosis inhibition to treat sepsis after acute alcohol exposure.

In our study, treatment with rhMFG-E8 significantly reduced circulating levels of endotoxin. Bacteremia and endotoxemia are increased in septic patients, reflecting hematogenic dissemination of bacteria from either the primary source of infection or secondary to increased gut permeability (Haussner et al. 2019). Bacteremia and endotoxemia are also increased in alcohol abusers because alcohol increases gut permeability (Enomoto et al. 2000). Interestingly, MFG-E8 is also produced by lamina propria macrophages in the small intestine and the colon (Aziz et al. 2009; Bu et al. 2007), where it promotes enterocyte homeostasis, restitution, proliferation, and basal-apical migration, all of which are crucial steps for intestinal mucosal healing (Bu et al. 2007; Mishiro et al. 2013; Kusunoki et al. 2015). Therefore, it is conceivable that rhMFG-E8 may have decreased endotoxin levels via its enterotrophic effects.

During sepsis, the systemic and local inflammatory responses are strongly driven by damage-associated molecular patterns (DAMPs), such as eCIRP and HMGB1, released by dying cells, as well as pathogen-associated molecular patterns (PAMPs), like endotoxin and peptidoglycan, released by bacteria. Indeed, treatment with rhMFG-E8 was associated not only with reduced presence of dying cells and endotoxin, but also with reduced serum levels of TNF-α and IL-6. Although anti-cytokine therapies have failed in clinical trials (Wesche-Soldato et al. 2007; Qin et al. 2006), systemic increases of proinflammatory cytokines have been associated with high sepsis mortality rates (Qin et al. 2006). The dysregulated inflammatory response in sepsis culminates in organ injury. Accordingly, in our study, treatment with rhMFG-E8 reduced the serum levels of the transaminases AST and ALT, suggesting attenuation of liver injury. It is important to indicate that alcohol also causes liver injury, and we have shown that the model of acute alcohol exposure (without sepsis) was associated with increased serum levels of AST (Wu et al. 2010). We also determined that rhMFG-E8 ameliorated sepsis-associated acute lung injury histologically. These findings are in agreement with previous observations in animals treated with rmMFG-E8 (Wu et al. 2010; Hansen et al. 2017a). A potential limitation of our study was the lack of hepatic, renal, and intestinal evaluations for histopathology and apoptosis. We would like to reiterate, however, that rmMFG-E8’s protective effects on systemic and organ-specific injuries in sepsis and pro-efferocytotic effects have already been well established and documented by our previously published studies (Wu et al. 2010; Cen et al. 2017; Miksa et al. 2006; Hansen et al. 2017a; Wu et al. 2012; Hansen et al. 2017b; Matsuda et al. 2012; Yang et al. 2015). Rather, the objective of our present study was to determine (A) whether the beneficial effect of rhMFG-E8 could be evaluated in a rat model of sepsis following after acute alcohol intoxication and (B) whether the benefit of rhMFG-E8 is equivalent to that of rmMFG-E8.

Although our prior study clearly demonstrated the beneficial effects of rmMFG-E8 in alcohol/sepsis, animal proteins are not used as therapeutic agents in humans due to immunogenicity concerns. Therefore, for the current project we generated and used rhMFG-E8. The molecular structure and the amino acid sequence of the human MFG-E8 is similar to the murine counterpart with the exception that the human MFG-E8 contains only one EGF-like domain and lacks the proline/threonine rich domain. Due to this partial homology, we were initially concerned about the adequacy of studying the effects of rhMFG-E8 in rodent model of sepsis and alcohol exposure. The present study shows, however, that not only rhMFG-E8 is just as effective in the rat as rmMFG-E8, but also suggests that rhMFG-E8 can adequately be evaluated using rat models of alcohol exposure and sepsis.

Our model of acute alcohol intoxication consisted of intravenous administration of alcohol for a total of 10 h, producing an average blood alcohol level of > 135 mg/dL without any lethality (Wu et al. 2010). Addition of CLP produced mortality in our alcohol/sepsis model starting 24 h after CLP. Treatment with rhMFG-E8 led to a significant improvement in the survival. A major drawback in our study is the model of combined alcohol exposure and sepsis. The model we used delivers alcohol intravenously to guarantee blood levels comparable to those of alcohol binge drinkers. As such it does not have oral intake of alcohol and, therefore, does not include its direct effects in the gut mucosal immunity. CLP alone causes severe sepsis with a minimal window for further aggravation of its complications, therefore a direct severity comparison between CLP alone and the combined model is not feasible. Finally, other limitations were that only one dose of rhMFG-E8 (20 μg/kg) was evaluated and that treatment was initiated at the beginning of alcohol exposure, preceding CLP by 10 h. In order to more firmly determine its therapeutic effects, future studies will need to include post-treatment of alcohol and sepsis with increasing doses of rhMFG-E8.

## Conclusion

In summary, the administration of rhMFG-E8 significantly ameliorates sepsis in the context of acute alcohol exposure. Thus, rhMFG-E8 should be further developed as a safe and effective treatment for septic patients who are also alcohol abusers.

## Data Availability

The datasets used and analyzed during the current study are available from the corresponding authors upon reasonable request.

## Abbreviations

ALT: Alanine aminotransferase
AMC: 7-amino-4-methylcoumarin
AST: Aspartate aminotransferase
BW: Body weight
CLP: Cecal ligation and puncture
EDTA: Ethylene diaminetetraacetic acid
EGF: Epidermal growth factor
EGTA: Ethylene glycol-bis(β-aminoethyl ether)-N,N,N′,N′-tetraacetic acid or egtazic acid
FITC: Fluorescein isothiocyanate
IACUC: Institutional Animal Care and Use Committee
IL-6: Interleukin 6
LAL: Limulus amebocyte lysate
LPS: Lipopolysaccharide
MFG-E8: Milk fat globule-epidermal growth factor-factor VIII
NIH: National Institutes of Health
PMSF: Phenylmethylsulfonyl fluoride
PS: phosphatidylserine
RGD: Arginine, glycine, and aspartic acid motif
rhMFG-E8: recombinant human milk fat globule-epidermal growth factor-factor VIII
rmMFG-E8: recombinant mouse milk fat globule-epidermal growth factor-factor VIII
TBS: Tris-buffered saline
TNF-α: Tumor necrosis factor alpha

## References

[CR1] Ajakaiye MA, Jacob A, Wu R, Yang WL, Nicastro J, Coppa GF, Wang P (2012). Recombinant human MFG-E8 attenuates intestinal injury and mortality in severe whole body irradiation in rats. PLoS One.

[CR2] Aloman C, Gehring S, Wintermeyer P, Kuzushita N, Wands JR (2007). Chronic ethanol consumption impairs cellular immune responses against HCV NS5 protein due to dendritic cell dysfunction. Gastroenterology.

[CR3] Annoni G, Weiner FR, Zern MA (1992). Increased transforming growth factor-beta 1 gene expression in human liver disease. J Hepatol.

[CR4] Aziz M, Brenner M, Wang P (2019). Extracellular CIRP (eCIRP) and inflammation. J Leukoc Biol.

[CR5] Aziz M, Jacob A, Matsuda A, Wang P (2011). Review: milk fat globule-EGF factor 8 expression, function and plausible signal transduction in resolving inflammation. Apoptosis.

[CR6] Aziz M, Yang WL, Wang P (2013). Measurement of phagocytic engulfment of apoptotic cells by macrophages using pHrodo succinimidyl ester. Curr Protoc Immunol.

[CR7] Aziz MM, Ishihara S, Mishima Y, Oshima N, Moriyama I, Yuki T, Kadowaki Y, Rumi MA, Amano Y, Kinoshita Y (2009). MFG-E8 attenuates intestinal inflammation in murine experimental colitis by modulating osteopontin-dependent alphavbeta3 integrin signaling. J Immunol.

[CR8] Bautista AP (1998). The role of Kupffer cells and reactive oxygen species in hepatic injury during acute and chronic alcohol intoxication. Alcohol Clin Exp Res.

[CR9] Bautista AP, Spitzer JJ (1999). Role of Kupffer cells in the ethanol-induced oxidative stress in the liver. Front Biosci.

[CR10] Bhatty M, Tan W, Basco M, Pruett S, Nanduri B (2017). Binge alcohol consumption 18 h after induction of sepsis in a mouse model causes rapid overgrowth of bacteria, a cytokine storm, and decreased survival. Alcohol.

[CR11] Bird MD, Kovacs EJ (2008). Organ-specific inflammation following acute ethanol and burn injury. J Leukoc Biol.

[CR12] Bu HF, Zuo XL, Wang X, Ensslin MA, Koti V, Hsueh W, Raymond AS, Shur BD, Tan XD (2007). Milk fat globule-EGF factor 8/lactadherin plays a crucial role in maintenance and repair of murine intestinal epithelium. J Clin Invest.

[CR13] Burnham EL, Moss M, Martin GS (2003). The effect of alcohol consumption on risk for sepsis and ARDS, in Intensive Care Medicine.

[CR14] Cen C, Aziz M, Yang WL, Zhou M, Nicastro JM, Coppa GF, Wang P (2017). Milk fat globule-epidermal growth factor-factor VIII attenuates sepsis-induced acute kidney injury. J Surg Res.

[CR15] Chaung WW, Jacob A, Ji Y, Wang P (2008). Suppression of PGC-1alpha by ethanol: implications of its role in alcohol induced liver injury. Int J Clin Exp Med.

[CR16] Cheyuo C, Jacob A, Wu R, Zhou M, Qi L, Dong W, Ji Y, Chaung WW, Wang H, Nicastro J, Coppa GF, Wang P (2012). Recombinant human MFG-E8 attenuates cerebral ischemic injury: its role in anti-inflammation and anti-apoptosis. Neuropharmacology.

[CR17] Cho YE, Yu LR, Abdelmegeed MA, Yoo SH, Song BJ (2018). Apoptosis of enterocytes and nitration of junctional complex proteins promote alcohol-induced gut leakiness and liver injury. J Hepatol.

[CR18] Chopra M, Sharma AC (2007). Apoptotic cardiomyocyte hypertrophy during sepsis and septic shock results from prolonged exposure to endothelin precursor. Front Biosci.

[CR19] Cook RT (1998). Alcohol abuse, alcoholism, and damage to the immune system--a review. Alcohol Clin Exp Res.

[CR20] de Wit M, Best AM, Gennings C, Burnham EL, Moss M (2007). Alcohol use disorders increase the risk for mechanical ventilation in medical patients. Alcohol Clin Exp Res.

[CR21] Enomoto N, Ikejima K, Bradford BU, Rivera CA, Kono H, Goto M, Yamashina S, Schemmer P, Kitamura T, Oide H, Takei Y, Hirose M, Shimizu H, Miyazaki A, Brenner DA, Sato N, Thurman RG (2000). Role of Kupffer cells and gut-derived endotoxins in alcoholic liver injury. J Gastroenterol Hepatol.

[CR22] Fleischmann C, Scherag A, Adhikari NK, Hartog CS, Tsaganos T, Schlattmann P, Angus DC, Reinhart K (2016). International forum of acute care T: assessment of global incidence and mortality of hospital-treated sepsis. Current estimates and limitations. Am J Respir Crit Care Med.

[CR23] Girardot T, Rimmele T, Venet F, Monneret G (2017). Apoptosis-induced lymphopenia in sepsis and other severe injuries. Apoptosis.

[CR24] Gustot T, Fernandez J, Szabo G, Albillos A, Louvet A, Jalan R, Moreau R, Moreno C (2017). Sepsis in alcohol-related liver disease. J Hepatol.

[CR25] Hanayama R, Tanaka M, Miwa K, Shinohara A, Iwamatsu A, Nagata S (2002). Identification of a factor that links apoptotic cells to phagocytes. Nature.

[CR26] Hansen LW, Khader A, Yang WL, Jacob A, Chen T, Nicastro JM, Coppa GF, Prince JM, Wang P (2017). Deficiency in milk fat globule-epidermal growth factor-factor 8 exacerbates organ injury and mortality in neonatal sepsis. J Pediatr Surg.

[CR27] Hansen LW, Yang WL, Bolognese AC, Jacob A, Chen T, Prince JM, Nicastro JM, Coppa GF, Wang P. Treatment with milk fat globule epidermal growth factor-factor 8 (MFG-E8) reduces inflammation and lung injury in neonatal sepsis. Surgery. 2017a;162:349–57 (PMC5513803). 10.1016/j.surg.2017.02.006 PMC5513803 28343695

[CR28] Hattori Y, Takano K, Teramae H, Yamamoto S, Yokoo H, Matsuda N (2010). Insights into sepsis therapeutic design based on the apoptotic death pathway. J Pharmacol Sci.

[CR29] Haussner F, Chakraborty S, Halbgebauer R, Huber-Lang M. Challenge to the intestinal mucosa during sepsis. Front Immunol. 2019;10:891 (PMC6502990). 10.3389/fimmu.2019.00891 PMC6502990 31114571

[CR30] Jin L, Batra S, Jeyaseelan S (2017). Diminished neutrophil extracellular trap (NET) formation is a novel innate immune deficiency induced by acute ethanol exposure in polymicrobial sepsis, which can be rescued by CXCL1. PLoS Pathog.

[CR31] Khanova E, Wu R, Wang W, Yan R, Chen Y, French SW, Llorente C, Pan SQ, Yang Q, Li Y, Lazaro R, Ansong C, Smith RD, Bataller R, Morgan T, Schnabl B, Tsukamoto H (2018). Pyroptosis by caspase11/4-gasdermin-D pathway in alcoholic hepatitis in mice and patients. Hepatology.

[CR32] Klingensmith NJ, Fay KT, Lyons JD, Chen CW, Otani S, Liang Z, Chihade DB, Burd EM, Ford ML, Coopersmith CM (2019). Chronic alcohol ingestion worsens survival and alters gut epithelial apoptosis and CD8+ T cell function after Pseudomonas aeruginosa pneumonia-induced sepsis. Shock.

[CR33] Kockara A, Kayatas M (2013). Renal cell apoptosis and new treatment options in sepsis-induced acute kidney injury. Ren Fail.

[CR34] Kusunoki R, Ishihara S, Tada Y, Oka A, Sonoyama H, Fukuba N, Oshima N, Moriyama I, Yuki T, Kawashima K, Ansary MM, Tajima Y, Maruyama R, Nabika T, Kinoshita Y (2015). Role of milk fat globule-epidermal growth factor 8 in colonic inflammation and carcinogenesis. J Gastroenterol.

[CR35] Lee WL (2018). Immunotherapy for sepsis: a good idea or another dead end?. Anesthesiology.

[CR36] Liuzzi JP, Narayanan V, Doan H, Yoo C (2018). Effect of zinc intake on hepatic autophagy during acute alcohol intoxication. Biometals.

[CR37] Luan YY, Yao YM, Xiao XZ, Sheng ZY (2015). Insights into the apoptotic death of immune cells in sepsis. J Interferon Cytokine Res.

[CR38] Matsuda A, Jacob A, Wu R, Aziz M, Yang WL, Matsutani T, Suzuki H, Furukawa K, Uchida E, Wang P (2012). Novel therapeutic targets for sepsis: regulation of exaggerated inflammatory responses. J Nippon Med Sch.

[CR39] Matsuda A, Jacob A, Wu R, Zhou M, Aziz M, Wang P (2013). Milk fat globule-EGF factor VIII ameliorates liver injury after hepatic ischemia-reperfusion. J Surg Res.

[CR40] Matsuda A, Wu R, Jacob A, Komura H, Zhou M, Wang Z, Aziz MM, Wang P (2011). Protective effect of milk fat globule-epidermal growth factor-factor VIII after renal ischemia-reperfusion injury in mice. Crit Care Med.

[CR41] McClain C, Barve S, Joshi-Barve S, Song Z, Deaciuc I, Chen T, Hill D (2005). Dysregulated cytokine metabolism, altered hepatic methionine metabolism and proteasome dysfunction in alcoholic liver disease. Alcohol Clin Exp Res.

[CR42] Mebazaa A, Laterre PF, Russell JA, Bergmann A, Gattinoni L, Gayat E, Harhay MO, Hartmann O, Hein F, Kjolbye AL, Legrand M, Lewis RJ, Marshall JC, Marx G, Radermacher P, Schroedter M, Scigalla P, Stough WG, Struck J, Van den Berghe G, Yilmaz MB, Angus DC (2016). Designing phase 3 sepsis trials: application of learned experiences from critical care trials in acute heart failure. J Intensive Care.

[CR43] Miksa M, Komura H, Wu R, Shah KG, Wang P (2009). A novel method to determine the engulfment of apoptotic cells by macrophages using pHrodo succinimidyl ester. J Immunol Methods.

[CR44] Miksa M, Wu R, Dong W, Das P, Yang D, Wang P (2006). Dendritic cell-derived exosomes containing milk fat globule epidermal growth factor-factor VIII attenuate proinflammatory responses in sepsis. Shock.

[CR45] Miksa M, Wu R, Dong W, Komura H, Amin D, Ji Y, Wang Z, Wang H, Ravikumar TS, Tracey KJ, Wang P (2009). Immature dendritic cell-derived exosomes rescue septic animals via milk fat globule epidermal growth factor-factor VIII [corrected]. J Immunol.

[CR46] Mishiro T, Kusunoki R, Otani A, Ansary MM, Tongu M, Harashima N, Yamada T, Sato S, Amano Y, Itoh K, Ishihara S, Kinoshita Y (2013). Butyric acid attenuates intestinal inflammation in murine DSS-induced colitis model via milk fat globule-EGF factor 8. Lab Investig.

[CR47] Moss M (2005). Epidemiology of sepsis: race, sex, and chronic alcohol abuse. Clin Infect Dis.

[CR48] Ness KJ, Fan J, Wilke WW, Coleman RA, Cook RT, Schlueter AJ (2008). Chronic ethanol consumption decreases murine Langerhans cell numbers and delays migration of Langerhans cells as well as dermal dendritic cells. Alcohol Clin Exp Res.

[CR49] O'Brien JM, Lu B, Ali NA, Martin GS, Aberegg SK, Marsh CB, Lemeshow S, Douglas IS (2007). Alcohol dependence is independently associated with sepsis, septic shock, and hospital mortality among adult intensive care unit patients. Crit Care Med.

[CR50] Oshima K, Yasueda T, Nishio S, Matsuda T. MFG-E8: origin, structure, expression, functions and regulation. In: Wang P, editor. MFG-E8 and Inflammation. Springer Dordrecht Heidelberg New York London; 2014. p. 1–32.

[CR51] Poon IK, Lucas CD, Rossi AG, Ravichandran KS (2014). Apoptotic cell clearance: basic biology and therapeutic potential. Nat Rev Immunol.

[CR52] Probst C, Parry CDH, Wittchen HU, Rehm J (2018). The socioeconomic profile of alcohol-attributable mortality in South Africa: a modelling study. BMC Med.

[CR53] Qiang X, Li J, Wu R, Ji Y, Chaung W, Dong W, Wang P (2011). Expression and characterization of recombinant human milk fat globule-EGF factor VIII. Int J Mol Med.

[CR54] Qiang X, Yang WL, Wu R, Zhou M, Jacob A, Dong W, Kuncewitch M, Ji Y, Yang H, Wang H, Fujita J, Nicastro J, Coppa GF, Tracey KJ, Wang P (2013). Cold-inducible RNA-binding protein (CIRP) triggers inflammatory responses in hemorrhagic shock and sepsis. Nat Med.

[CR55] Qin S, Wang H, Yuan R, Li H, Ochani M, Ochani K, Rosas-Ballina M, Czura CJ, Huston JM, Miller E, Lin X, Sherry B, Kumar A, Larosa G, Newman W, Tracey KJ, Yang H (2006). Role of HMGB1 in apoptosis-mediated sepsis lethality. J Exp Med.

[CR56] Sabha BH, Alzahrani F, Almehdar HA, Uversky VN, Redwan EM (2018). Disorder in milk proteins: lactadherin multifunctionality and structure. Curr Protein Pept Sci.

[CR57] Scaffidi P, Misteli T, Bianchi ME (2002). Release of chromatin protein HMGB1 by necrotic cells triggers inflammation. Nature.

[CR58] Seeley EJ, Bernard GR (2016). Therapeutic targets in sepsis: past, present, and future. Clin Chest Med.

[CR59] Shah KG, Wu R, Jacob A, Molmenti EP, Nicastro J, Coppa GF, Wang P (2012). Recombinant human milk fat globule-EGF factor 8 produces dose-dependent benefits in sepsis. Intensive Care Med.

[CR60] Simou E, Leonardi-Bee J, Britton J (2018). The effect of alcohol consumption on the risk of ARDS: a systematic review and meta-analysis. Chest.

[CR61] Singer M, Deutschman CS, Seymour CW, Shankar-Hari M, Annane D, Bauer M, Bellomo R, Bernard GR, Chiche JD, Coopersmith CM, Hotchkiss RS, Levy MM, Marshall JC, Martin GS, Opal SM, Rubenfeld GD, van der Poll T, Vincent JL, Angus DC (2016). The Third International Consensus definitions for sepsis and septic shock (Sepsis-3). JAMA.

[CR62] Smith CC, Guevremont D, Williams JM, Napper RM (2015). Apoptotic cell death and temporal expression of apoptotic proteins Bcl-2 and Bax in the hippocampus, following binge ethanol in the neonatal rat model. Alcohol Clin Exp Res.

[CR63] Substance Abuse and Mental Health Services Administration (2017). Key substance use and mental health indicators in the United States: results from the 2016 National Survey on Drug Use and Health Center for Behavioral Health Statistics and Quality, Substance Abuse and Mental Health Services Administration.

[CR64] Tilg H, Wilmer A, Vogel W, Herold M, Nolchen B, Judmaier G, Huber C (1992). Serum levels of cytokines in chronic liver diseases. Gastroenterology.

[CR65] Trevejo-Nunez G, Kolls JK, de Wit M (2015). Alcohol use as a risk factor in infections and healing: a clinician's perspective. Alcohol Res.

[CR66] Vincent JL, Sakr Y (2019). Clinical trial design for unmet clinical needs: a spotlight on sepsis. Expert Rev Clin Pharmacol.

[CR67] Waldschmidt TJ, Cook RT, Kovacs EJ (2008). Alcohol and inflammation and immune responses: summary of the 2006 Alcohol and Immunology Research Interest Group (AIRIG) meeting. Alcohol.

[CR68] Wesche-Soldato DE, Swan RZ, Chung CS, Ayala A (2007). The apoptotic pathway as a therapeutic target in sepsis. Curr Drug Targets.

[CR69] Wu R, Chaung WW, Zhou M, Ji Y, Dong W, Wang Z, Qiang X, Wang P (2010). Milk fat globule EGF factor 8 attenuates sepsis-induced apoptosis and organ injury in alcohol-intoxicated rats. Alcohol Clin Exp Res.

[CR70] Wu R, Dong W, Wang Z, Jacob A, Cui T, Wang P (2012). Enhancing apoptotic cell clearance mitigates bacterial translocation and promotes tissue repair after gut ischemia-reperfusion injury. Int J Mol Med.

[CR71] Yang WL, Sharma A, Zhang F, Matsuo S, Wang Z, Wang H, Wang P (2015). Milk fat globule epidermal growth factor-factor 8-derived peptide attenuates organ injury and improves survival in sepsis. Crit Care.

[CR72] Yasueda T, Oshima K, Nakatani H, Tabuchi K, Nadano D, Matsuda T (2015). A protective effect of milk fat globule EGF factor VIII (MFG-E8) on the spontaneous fusion of milk fat globules in breast milk. J Biochem.

[CR73] Zhang F, Shah KG, Qi L, Wu R, Barrera R, Nicastro J, Coppa GF, Wang P (2012). Milk fat globule epidermal growth factor-factor 8 mitigates inflammation and tissue injury after hemorrhagic shock in experimental animals. J Trauma Acute Care Surg.

